# Annotations

**Published:** 1897-05-15

**Authors:** 


### ANNOTATIONS.

### Scurvy.

WE are so accustomed to think of scurvy in association with long sea voyages and arctic expeditions that it may come as a surprise to some to hear that one of the troubles of the troops in Matabeleland last year arose from a condition which one can hardly doubt was but a variety of that disease. Dr. R. T. Mitchell, who was lately surgeon to the British South African Company's forces, writing on the diseases which occurred among the troops under his care, says that among the minor ailments the so-called "Veldt Sores" gave more trouble than any other. "Whenever a man scratched himself with thorns or otherwise lacerated the skin there would appear one or more persistent eczematous ulcers." The condition was practically universal among the troops engaged, affecting both officers and men. On investigation it turned out that this condition was intimately connected with a scurvy-like condition of the blood, which in turn was connected with the diet supplied to the troops. The difficulties of transport were great; rinderpest had killed most of the cattle; and in the diet served out vegetables were not included. So soon as lime-juice was supplied more regularly and compressed vegetables were more generally served in the diet, the disease began to disappear. As proof that these sores were connected with scurvy, it is stated that not only did recovery take place under an anti-scorbutic dietary, but that among those who suffered from them sponginess of the gums, with bleeding and loosening of the teeth, and with other forms of hæmorrhage, occurred far too frequently to be explained on any other hypothesis than that of their being of a scorbutic nature.

### Medical Teaching in Germany.

So much of our knowledge in the field of medicine hails from Germany, and German literature is so abundant and ever-flowing a source of fact and theory, that it is not without interest to note some of the peculiarities of German hospitals and schools as they appeared to a modern American observer, Dr. Leon Solomon, of Kentucky. The whole system on which English voluntary hospitals are conducted hinges entirely on a recognition of the fact that their first duty is the cure of the sick. In Germany, however, a somewhat different view seems to be taken of the situation. Dr. Solomon says: "The comforts of the patients, except in private institutions, are, as a rule, not materially cared for. The one and sole aim seems to be to make the diagnosis; even treatment is regarded as a secondary matter. Each hospital prides itself upon its laboratories, and, I might say, in a marked degree upon its 'dead house.' The royal government lends a protecting hand and the hospital never wants for scientific apparatus and working paraphernalia. Coupled with this comes the German patient, who, accustomed to being ruled, accustomed to army discipline and subjection, makes a very desirable subject for medical attention." Speaking of the extremely methodical and complete examination of every part of the body which is customary before any diagnosis is attempted, he goes on to say, "the treatment is a secondary consideration with the Germans. They exhaust every avenue that the nature of the case may be solved, and are then quite satisfied to let the therapy take care of itself." In regard to methods of teaching, the great subdivision of the subjects is a point of interest. A distinction is drawn between the surgeon—the man of knowledge, the clear talker, the careful demonstrator—and the operator, a man of technique, whose well-drilled assistants, "work together like an intricate yet perfect piece of machinery." Clinical work, moreover, is not restricted to the cases which happen to be in the hospital. Everything is subservient to teaching. Patients suffering from chronic ailments are tracked and kept note of, so that when the professor is ready to lecture on any special subject, illustrative cases are brought up for examination and demonstration. Lastly, it is to be noted, that in Germany the didactic lecture has almost been done away with; every disease is illustrated by a clinical case, or rather by a series of them.

### Typhoid Fever in Villages.

A STUDY of the reports on local epidemics issued from time to time by the Local Government Board should increase our respect for the saying of St. Paul, that we stand in jeopardy every hour. People are far too ready to think that because their little district has for years remained exempt from serious epidemics such exemption will continue, and everywhere we find them "putting up with" insanitary conditions on the plea that hitherto they have done no harm. These good folk forget that for the production of such a disease as typhoid fever foul living is but the precedent cause which makes the outbreak possible; and that if they live foully, however long they may escape, they will surely suffer so soon as infection is introduced. Kessingland is a little parish near Lowestoft with about 1,200 inhabitants. Of proper sewerage there is none. Slop water finds its way into brooks and watercourses, and some of the larger houses drain into leaky cesspools. Privy-pits are the rule. Into them the surface water finds its way and then soaks into the surrounding soil, and all the time the whole of the water supply of the place is derived from surface wells, some of which are within a few feet of the cesspools. Yet among all these foul surroundings the people lived, and apparently were healthy. Between 1889 and 1893 only four cases of typhoid fever were recorded, two of which occurred in 1893. But from the beginning of 1894 to October 31st, 1896, there were seventy-nine cases. The fact is, that in 1893 a case was imported into a specially foul quarter of the district infecting certain wells, around which the majority of the subsequent cases occurred. One of these wells, about fifteen feet deep, had within a radius of twenty yards of it no less than ten privy-pits, and all of them received the soakage from gardens heavily manured with the contents of similar receptacles of filth. That these villagers should suffer from typhoid is a matter of no particular interest; an outbreak of the sort was sure to occur sooner or later. What is of interest is that such a district should remain comparatively free for so long even amid all its filth, and the proof so afforded of the fact that no community can safely trust to its freedom from typhoid as a test of its sanitary surroundings. There are certain well-known conditions in which typhoid fever is sure to take root if it be but introduced, and when those conditions are present the people go in jeopardy all their lives, however good may be their ecords of disease.

